# OLDER AGE IS ASSOCIATED WITH TEMPORAL DISCOUNTING OF TIME USE BUT NOT MONETARY REWARDS

**DOI:** 10.1093/geroni/igad104.2134

**Published:** 2023-12-21

**Authors:** Yochai Shavit, Brett Anderson, Laura Carstensen

**Affiliations:** Stanford University, Stanford, California, United States; MemorialCare Long Beach Medical Center, Anaheim, California, United States; Stanford, Stanford, California, United States

## Abstract

Age differences in temporal discounting have long puzzled researchers. Although older adults tend to prioritize the present over the future due to more limited time horizons compared to younger adults there is no evidence for an age association with temporal discounting of monetary rewards. Socioemotional selectivity theory posits that as time horizons become more limited goals related to emotional meaning are prioritized over future-oriented goals because they are realized in the present, leading older adults to place more value on experiences than younger adults. A small body of evidence showing age-related preferences for small, immediate, rewards in emotionally meaningful domains is consistent with SST. However, prior studies used hypothetical tasks with vague trade-offs, limiting their interpretability and generalizability. In the present study, we developed a novel paradigm to examine age differences in temporal discounting of rewards related to emotional experience in a controlled environment. 120 participants, aged 22-96 years, came to the lab and made a series of choices indicating if they would prefer to replace 5 minutes of boring tasks with an emotionally meaningful variant of the task today, or a larger amount of time in their next visit in six months. Participants then made similar choices about the timing of receiving comparable monetary rewards. We hypothesized that in contrast to monetary rewards, older adults are less likely than younger adults to wait for rewards related to emotional experiences because time becomes increasingly valued as it grows scarce. Findings support the hypothesis and highlight the role of perceived time-horizons.

